# Rheological Performance of Wholemeal Flour from Bread Wheat Under Contrasting Agro-Ecological Conditions: A Multi-Environment Analysis of Mixolab^®^ 2 Parameters and Genotypic Stability

**DOI:** 10.3390/foods15183238

**Published:** 2026-09-14

**Authors:** Hind El Bouzidi, Mona Taghouti, Aziz Baidani, Fatima Gaboun, Degu Wuletaw Tadesse, Ilyass Britel, Mackaye Moussa Hassane, Mohamed Amine Abdellaoui, Imane El Ftouh, Fatima Ezzahra Oudrhiri, Ghizlane Diria

**Affiliations:** 1 Faculty of Sciences and Techniques, Hassan I University, Settat 26000, Morocco; elbouzidi.h.fst@uhp.ac.ma (H.E.B.); aziz.baidani@uhp.ac.ma (A.B.); 2 National Institute of Agronomic Research, Rabat 10112, Morocco; mouna.taghouti@inra.ma (M.T.); fatima.gaboun@inra.ma (F.G.); 3 International Center for Agricultural Research in the Dry Areas (ICARDA), Rabat Instituts, Rabat 10090, Morocco; w.tadesse@cgiar.org; 4 Faculty of Science, Mohammed V University, Rabat 10112, Morocco; ilyass_britel@um5.ac.ma (I.B.);; 5 Faculty of Science, Ibn Tofail University, Kenitra 14000, Morocco; mohamedamine.abdellaoui@uit.ac.ma; 6 Hassan II Institute of Agronomy and Veterinary Medicine (IAV Hassan II), Rabat 10112, Morocco; imane.elftouh@inra.ma (I.E.F.); fatimazahra.oudrhiri@inra.ma (F.E.O.)

**Keywords:** bread wheat, Mixolab^®^ 2, genotype × environment interaction, rheological parameters

## Abstract

Bread wheat technological quality is jointly influenced by genotype, environment, and their interaction (G × E). Although Mixolab^®^ has been used to characterize rheological variation among wheat genotypes, its combined application with stability analysis remains limited in Moroccan multi-environment trials. Rheological variability and relative stability were evaluated using wholemeal flour from 13 bread wheat varieties grown across three contrasting agro-ecological zones in Morocco. Under a fixed hydration level of 60%—rather than the sample-specific hydration used in the standard ICC 173 procedure—nine Mixolab^®^ 2 parameters (C1–C5, α, β, γ, and mixing stability time) were measured. Two-way ANOVA, REML-based BLUPs, principal component analysis (PCA), and inter-environment stability indices were applied to evaluate varietal performance and relative stability. Highly significant effects (*p* < 0.001) of genotype, environment, and G × E were observed for all parameters. Under fixed hydration, the observed responses may reflect differences in flour composition, water-binding properties, and dough development at a common water level. Environmental contributions were largest for α and mixing stability time, whereas genotype-associated contributions were largest for C4 and C5. The first two principal components explained 72.6% of the total variability and identified three rheological groups under fixed hydration conditions: low-consistency genotypes, varieties with balanced rheological profiles, and starch-oriented genotypes. Within the three-environment, single-season network, Kenz, Najia, Khadija, Malika, and Ibtissam showed the lowest inter-environment variation, whereas Lina and Irchad showed the greatest. Whether these rankings remain consistent under the standard ICC 173 sample-specific hydration procedure remains unknown. These results illustrate the potential of combining Mixolab^®^ 2 parameters with mixed-model and stability analyses to characterize rheological patterns under fixed hydration conditions. Additional environments and cropping seasons are required before broad adaptation or environment-specific recommendations can be made.

## 1. Introduction

Bread wheat (*Triticum aestivum* L.) is one of the most important cereal crops worldwide, contributing significantly to global food security and the agri-food industry. Wheat flour technological quality, a key determinant of processing performance and end-product quality, is governed by complex interactions between genetic and environmental factors [1]. This property can be effectively assessed by rapid and accurate rheological methods capable of capturing the total complexity of dough behavior.

Determining the rheological and thermal properties of wheat flours is also a major challenge for the milling and baking industries. The Mixolab^®^ 2 records dough consistency during mixing, heating, and cooling, providing indirect information on protein- and starch-related behavior [2]. The instrument generates a composite curve characterized by nine parameters (C1–C5, stability time, α, β, and γ). These parameters describe dough stability and torque changes associated with protein weakening, starch gelatinization, amylolytic activity, and starch retrogradation [3]. Previous studies have reported associations between Mixolab^®^ parameters and breadmaking characteristics, including relationships between C1, bread volume, and crumb structure [4].

Mixolab is now widely used as a tool to determine the rheological behavior of wheat flours. It provides an analytical understanding of the characteristics of dough during kneading, heating and cooling within a single analytical process. It provides information on gluten and starch as it records protein weakening as well as the gelatinization of starch. The technological quality of bread wheat flours has been successfully assessed in relation to their baking properties using Mixolab^®^ [5]. It has been applied to distinguish between wheat varieties in India under thermal stress with regard to starch and protein functions [6]. Similarly, studies conducted in Italy have demonstrated its usefulness for characterizing common and durum wheat intended for industrial processing [7,8,9]. These studies confirm the relevance of Mixolab^®^ for relating flour technological performance to biochemical composition while considering genotype and environmental effects.

However, the effects of environmental variation on these rheological parameters remain insufficiently characterized. This is mainly due to genotype × environment interaction, which corresponds to changes in the phenotypic expression of genotypes under different environmental conditions [10]. Environmental effects can lead to differential varietal responses across regions, resulting in changes in the expression of technological quality traits. Recent research indicates that environmental influences can account for up to 65% of the total phenotypic variance of certain quality traits, which highlights the need to conduct a multi-environment evaluation [11].

Although previous studies have assessed Mixolab^®^ parameters across wheat genotypes and experimental or climatic conditions, including Indian wheat varieties [6], an Italian field study and a review of durum wheat rheology [7,8], and an assessment of climatic effects on durum wheat quality [9], the integration of these parameters with BLUP-based predictions and explicit stability analysis across all nine Mixolab^®^ 2 parameters has received limited attention in Moroccan multi-environment trials.

This methodological gap limits the effective integration of rheological data into breeding programs and reduces their usefulness for predicting quality performance across diverse environmental conditions. Climate variability and environmental constraints are major limitations to wheat production in Mediterranean and semi-arid conditions, directly affecting grain quality and technological characteristics. Climate change strengthens genotype × environment interactions, complicating both varietal selection and the prediction of quality traits across environments. Morocco, with its diverse agro-ecological gradient spanning semi-arid lowlands to humid mountainous areas, represents a relevant model system for investigating such interactions on wheat rheological traits, with potential relevance for future wheat quality research in comparable Mediterranean and semi-arid environments [12,13].

Accordingly, this study presents a regional multi-environment rheological characterization of bread wheat using the Mixolab^®^ 2, combining ANOVA, mixed linear models, BLUPs, and multivariate analyses. We hypothesized that (i) the starch-related parameters C3, C4, and C5 would show larger genotype-associated contributions to variation than C1, C2, and stability time under fixed hydration conditions, whereas the latter parameters would show greater environmental sensitivity, and (ii) varieties showing lower inter-environment variation would exhibit more stable protocol-dependent rheological profiles within the evaluated environmental network. To test these hypotheses, thirteen bread wheat varieties were evaluated in three contrasting agro-ecological environments. The objectives were to quantify genotype × environment interactions for the nine Mixolab^®^ 2 parameters, identify varieties showing comparatively stable rheological responses under fixed hydration within the tested environmental network, and assess the potential of these parameters for preliminary, protocol-dependent wheat quality comparisons across growing conditions.

## 2. Materials and Methods

### 2.1. Plant Material and Experimental Design

The study included thirteen Moroccan bread wheat varieties (Table 1) evaluated at three INRA experimental stations: Sidi El Aydi (SEA), Merchouch (MCH), and Annoceur (ANN). These sites represent contrasting agro-ecological conditions, ranging from semi-arid lowlands (SEA and MCH) to humid mountainous environments (ANN) (Table S1), and cover a climatic and altitudinal gradient representative of Moroccan wheat-growing areas.

The plant material evaluated in this study was obtained from field trials conducted during the 2023/2024 cropping season. The trials were established in a randomized complete block design (RCBD) with three replications at each experimental site. Detailed site-specific information on harvested plot area, sowing and harvest dates, crop management, water regime, climatic conditions, grain yield and post-harvest handling is provided in Supplementary Table S1. Crop management practices followed the local agronomic recommendations for each experimental site.

### 2.2. Wholemeal Flour Preparation

Wheat samples were ground into wholemeal flour using a Cyclone Sample Mill equipped with a 0.5 mm screen. No subsequent sieving or separation of the bran, germ, and endosperm fractions was performed; therefore, all rheological measurements were conducted on wholemeal flour. After milling, the moisture content of each wholemeal flour sample was determined and entered into the Mixolab^®^ software. The software automatically calculated the required flour test portion on a 14% moisture basis; the samples were not physically conditioned to 14% moisture before testing.

### 2.3. Mixolab^®^ Rheological Analysis

The rheological properties of the wholemeal flour samples were evaluated using a Mixolab^®^ 2 instrument (Chopin Technologies, Villeneuve-la-Garenne, France). The mixing and temperature profile of ICC Method 173 was followed, except for water addition. Rather than adjusting hydration separately for each sample to obtain the standard C1 torque of 1.10 N·m, all samples were analyzed at a fixed hydration level of 60% on a 14% moisture basis. This modified protocol was selected to compare the relative dough consistency of all genotype–environment combinations under the same hydration level. A constant-hydration Mixolab^®^ protocol using 60% water on a 14% moisture basis has previously been applied to wheat flour formulations [14]. Because its flour matrix and experimental objective differed from those of the present study, the study is cited only as a methodological precedent.

Under this protocol, C1 was allowed to vary and was considered an experimental response reflecting dough consistency at 60% hydration rather than a standardized value of 1.10 N·m. Stability time was consequently interpreted as the duration for which this protocol-dependent consistency was maintained. Although the same hydration level was applied to all samples, differences in flour composition and water-binding capacity may affect the amount of water available for protein development, starch gelatinization, enzymatic activity and starch retrogradation. Therefore, C1, stability time and C2–C5 were interpreted as relative responses within the present dataset. The absolute Mixolab^®^ values obtained in this study should not be considered directly equivalent to those generated using the standard variable hydration procedure unless the difference in hydration protocol is explicitly taken into account.

The Mixolab^®^ continuously measures dough consistency (N·m) under simultaneous mechanical and thermal stresses. This enables the behavior of the major flour components, particularly proteins and starch, to be monitored during mixing, heating and cooling. The resulting Mixolab^®^ curve provides nine principal parameters. C1 (N·m) corresponds to the maximum dough consistency reached during mixing. Dough stability (min) was automatically calculated via Mixolab^®^ software as the duration of the constant-temperature mixing phase during which the upper envelope of the torque curve remained above 89% of the C1 value, where C1 corresponds to the maximum torque measured for each individual curve. The standard relative definition remained applicable under fixed hydration, although the resulting stability values were interpreted as protocol-dependent indicators of relative mixing tolerance [15]. C2 (N·m) corresponds to the minimum torque reached during protein weakening under the combined effects of mechanical shear and increasing temperature. C3 (N·m) represents the maximum torque associated with starch gelatinization as starch granules absorb water and swell during heating. C4 (N·m) represents the torque recorded during the high-temperature phase and describes hot-gel consistency, whereas C5 (N·m) represents the increase in consistency observed during cooling [9]. The α, β and γ slopes (N·m·min^−1^) describe the rates of protein weakening between C1 and C2, starch gelatinization between C2 and C3, and enzymatic degradation between C3 and C4, respectively. A more negative α value indicates faster protein weakening, whereas β and γ describe the kinetics of starch gelatinization and hot-gel breakdown, respectively [2,16].

### 2.4. Statistical Analyses

Each station was considered a separate environment. Statistical analyses were performed in R version 4.4.2 using the lme4, agricolae, FactoMineR, and factoextra packages. For each Mixolab^®^ parameter, the effects of genotype, environment and their interaction were evaluated using the following fixed-effects model:

Y_ijk_ = μ + G_i_ + E_j_ + (GE)_ij_ + ε_ijk_

where (Yijk) is the recorded value for genotype (i) in environment (j) for observation k, μ is the overall mean, (Gi) is the genotype effect, (Ej) is the environment effect, ((GE)ij) is the genotype × environment interaction, and (εijk) is the residual term. Genotype, environment and genotype × environment interaction were considered fixed effects. The analytical dataset contained 117 observations, corresponding to 13 genotypes evaluated in three environments, with three observations per genotype–environment combination. The degrees of freedom were 12 for genotype, 2 for environment, 24 for genotype × environment interaction, 78 for residual error, and 116 in total. A similar partitioning of wheat quality variation into genotype, environment, and genotype × environment components has been applied to processing-quality traits [17].

For each source of variation, the F-statistic was calculated by dividing its mean square by the residual mean square. Effect magnitude was quantified using eta-squared (η^2^), calculated by dividing the sum of squares attributable to each source by the total corrected sum of squares. The percentage contribution of each source was obtained by multiplying the resulting eta-squared value by 100. The total corrected sum of squares included the sums of squares attributable to genotype, environment, genotype × environment interaction and residual error.

For BLUP estimation, the model was written as follows:

Y_ijk_ = μ + E_j_ + g_i_ + (gE)_ij_ + ε_ijk_

where environment was treated as a fixed effect, whereas genotype and genotype × environment interaction were considered random effects. The random terms were assumed to follow independent normal distributions, with the following:

g_i_∼N (0, σG^2^);

(gE)_ij_∼N (0, σGE^2^);

and ε_ijk_∼N (0, σe^2^).

Variance components and BLUPs were estimated using restricted maximum likelihood. Residuals were assumed to be independent and normally distributed with a mean of zero and constant variance across genotype–environment combinations. Although the field trials were established using a randomized complete block design, block identity was not retained as a separate factor in the rheological analytical dataset. Consequently, block effects were not estimated independently, and the corresponding unexplained variability was incorporated into the residual term. This limitation was considered when interpreting genotype × environment interactions and genotypic stability. The genotype and genotype × environment BLUPs were used to evaluate average genotypic performance and relative responses across environments. The BLUPs were standardized within each environment using z-scores and subsequently subjected to principal component analysis and hierarchical clustering using Ward’s method and Euclidean distances. The optimal number of clusters was evaluated using silhouette width and gap statistics. Inter-environment stability was quantified for each genotype as the difference between the maximum and minimum standardized genotype × environment BLUPs across the three environments. Lower values indicate smaller variation across the evaluated environments and, therefore, greater relative stability within the tested environmental network. This interpretation is consistent with the general concept of phenotypic stability described by [18]. Given the limited number of environments and the evaluation of a single cropping season, this index was interpreted as a preliminary measure of relative stability rather than definitive evidence of broad adaptation. Pearson correlations among rheological parameters were calculated, and the resulting *p*-values were adjusted using the false discovery rate procedure.

## 3. Results and Discussion

### 3.1. Genotype, Environment and Genotype × Environment Effects

The combined ANOVA revealed highly significant effects of genotype (G), environment (E) and genotype × environment interaction (G × E) on all nine Mixolab^®^ parameters (*p* < 0.001; Table 2). Their relative contributions nevertheless differed among parameters. Genotype contributed most strongly to C3 (32.1%), C4 (35.2%) and C5 (43.8%), whereas environment made the largest contribution to dough stability (33.8%) and α (42.1%). G × E was the principal source of variation for C1 (32.3%), C2 (29.3%), β (50.8%) and γ (38.3%), although the contributions of E and G × E to C1 were nearly identical. This trait-dependent partitioning agrees with previous studies showing that genotype, growing environment and their interaction can differentially affect wheat rheological and processing-quality traits [9,11,19]. C3–C5 and the β and γ slopes describe torque changes during the Mixolab^®^ heating–cooling cycle and are commonly discussed in relation to starch gelatinization, hot-paste stability and retrogradation-related behavior [5,20]. However, they remain indirect rheological indicators rather than direct measurements of starch composition or α-amylase activity. Moreover, the environment term represents a composite contrast among MCH, ANN and SEA and does not separate the individual effects of climate, soil, water regime or crop management. The significant G × E effects therefore support the subsequent use of BLUP and multivariate analyses to investigate genotypic stability and environment-specific responses.

The fixed hydration protocol must be considered when interpreting the observed differences among genotypes and environments. Unlike the standard ICC 173 procedure, in which water addition is adjusted to obtain a C1 torque of 1.10 N·m, C1 was allowed to vary in the present study and therefore represents dough consistency at 60% hydration. Consequently, a higher C1 value cannot be interpreted independently as evidence of greater water absorption capacity or intrinsic gluten strength, because it reflects the combined effects of flour composition, water-binding capacity and dough resistance under the applied hydration condition. Stability time is likewise conditional on the initial consistency obtained at 60% hydration. Although the same hydration level was applied to all samples, differences in their water-binding capacity may have altered the amount of free water available for protein weakening, starch gelatinization, enzymatic activity, and retrogradation, thereby influencing C2–C5. Accordingly, the differences and genotype × environment patterns observed here should be interpreted as relative comparisons among samples analyzed under the same protocol. The absolute values should not be compared directly with those reported in studies using hydration adjusted to C1 = 1.10 N·m [21,22].

Mixolab^®^ parameters were interpreted here as operational rheological descriptors. Because protein content, wet gluten, glutenin/gliadin composition, SDS sedimentation, damaged starch, amylose content, Falling Number and α-amylase activity were not measured, the biochemical mechanisms discussed below should be regarded as literature-based hypotheses rather than as mechanisms demonstrated directly by the present data. Consequently, the results are primarily described in terms of the measured torque and slope responses.

The station-level comparison revealed distinct rheological patterns (Table 3). MCH exhibited the highest mean C1, C2 and C5 responses but the shortest stability, indicating greater initial and final dough consistency accompanied by lower mixing tolerance under fixed hydration. This interpretation is consistent with the use of C1 and stability as indicators of dough consistency and mixing behavior in previous Mixolab^®^ studies [2,5,6,21]. SEA showed the longest stability and the highest mean C4 and β responses. In contrast, ANN exhibited the lowest mean C1, C2, C4 and C5 responses, although its mean C3 value was slightly higher than those recorded at MCH and SEA. Similar effects of growing environment and climatic conditions on wheat thermo-mechanical behavior have been reported previously [7,9,11,19].

The principal genotype contrasts further demonstrated that the Mixolab^®^ parameters did not respond uniformly across environments. At MCH, Lina combined the highest C1 and C5 responses with the shortest stability, whereas Ibtissam combined the lowest C1 response with the longest stability. At SEA, Snina exhibited the highest C3–C5 responses, while Bandera showed the lowest C1–C5 responses despite relatively long stability. At ANN, Irchad combined the highest C1 response with the lowest C3–C5 responses, whereas Snina maintained the highest C3–C5 responses and Achtar showed the longest stability. The C3–C5 parameters are generally associated with starch gelatinization, hot-paste stability and retrogradation-related changes during the Mixolab^®^ heating–cooling cycle [5,6,21]. However, these responses should be interpreted as functional rheological indicators rather than direct measurements of starch composition or enzymatic activity. Overall, the contrasting varietal responses across stations support the significant genotype × environment effects detected by ANOVA and are consistent with previous multi-environment wheat quality studies [9,11,19]. Complete genotype-specific means and statistical comparisons are provided in Supplementary Tables S2–S4.

**Figure 1:**
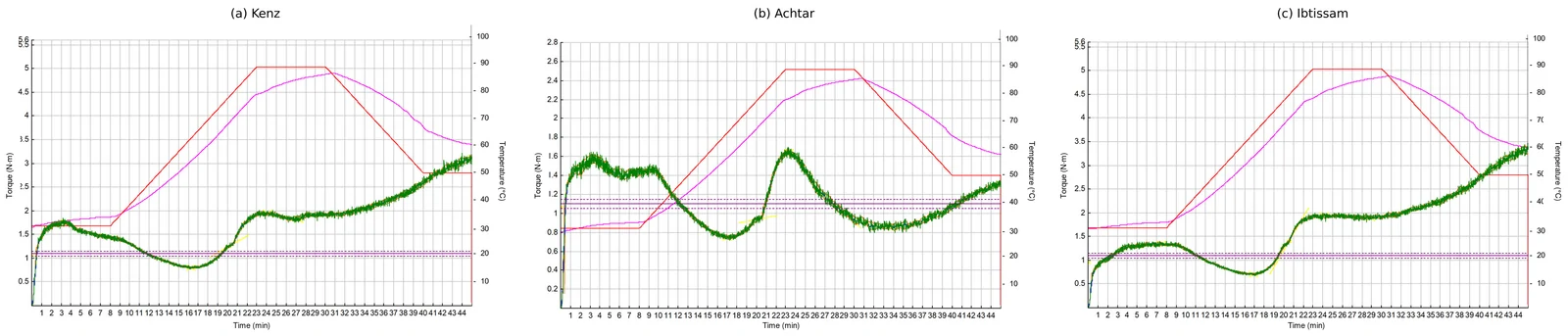
Mixolab^®^ 2 rheological profiles of Kenz (**a**), Achtar (**b**), and Ibtissam (**c**) at the Annoceur (ANN) station. The green curves represent dough torque (N·m; left y-axis), the red lines represent the programmed Mixolab temperature profile, and the magenta lines represent dough temperature (°C; right y-axis). The horizontal reference lines are software-generated guides and were not used to standardize C1 or calculate stability under the fixed 60% hydration protocol.

To illustrate contrasting varietal responses under humid mountainous conditions, representative curves were presented for Kenz, Achtar and Ibtissam at Annoceur (ANN) (Figure 1). Achtar showed comparatively lower C4 and C5 responses, whereas Kenz displayed a more balanced curve across the measured Mixolab^®^ parameters. Ibtissam exhibited comparatively elevated C3–C5 responses, reflecting a starch-oriented rheological profile under the applied protocol. These curves provide descriptive examples of the contrasting responses observed at ANN and should not be interpreted as evidence of broad adaptation, biochemical composition or end-product performance.

### 3.2. Relationships Among Rheological Parameters

Pearson correlation analysis revealed significant relationships among Mixolab^®^ 2 parameters (Table S5). Under fixed hydration conditions, C1 was positively correlated with C2 (r = 0.72; *p* < 0.001), showing that samples with greater initial dough consistency generally retained higher torque during heating. This relationship should be interpreted within the present fixed hydration protocol and not as direct evidence of intrinsic gluten strength.

In addition, the starch-related parameters C3, C4 and C5 were positively correlated with one another (r = 0.40–0.76; *p* < 0.001), reflecting the interdependence of starch gelatinization, hot-gel stability and retrogradation. This finding is consistent with previous evidence showing that starch molecular structure influences its thermal behavior, particularly gelatinization and retrogradation [23]. Significant negative correlations were observed between C1 and α (r = −0.66; *p* < 0.001) and between C2 and α (r = −0.60; *p* < 0.001). Because more negative α values represent faster protein weakening, these relationships indicate that samples with higher C1 and C2 torques also tended to exhibit steeper protein-weakening slopes. They should therefore not be interpreted independently as evidence of greater gluten thermal tolerance.

Stability time was positively correlated with α (r = 0.37; *p* < 0.001) and β (r = 0.31; *p* < 0.001). The positive relationship with α indicates that longer mixing stability was associated with less negative α values and therefore slower protein weakening, whereas the association with β suggests a relationship between mixing behavior and subsequent starch-gelatinization kinetics. The γ slope showed relatively weak correlations with C3 (r = −0.25; *p* < 0.01) and C4 (r = 0.21; *p* < 0.05), indicating a limited association between starch-gel consistency and the rate of hot-gel breakdown. Overall, these correlations help characterize the relative rheological profiles of the varieties under the present experimental conditions, although direct baking tests would be required to confirm their implications for breadmaking performance.

### 3.3. Genotypic Performance and Genotype × Environment Responses

All genotype and genotype × environment BLUP patterns reported below refer specifically to responses measured under the fixed 60% hydration protocol and should not be interpreted as protocol-independent varietal rankings. Genotypic performance was estimated using genotype BLUPs (BLUP-G). The BLUP-G (Table S6) values varied among genotypes for all Mixolab^®^ parameters, with the largest ranges observed for stability (3.60 units) and C5 (1.02 units), indicating marked differences in predicted responses under fixed 60% hydration. Regarding C1 and C2 responses, Lina showed positive BLUPs for C1 (+0.195) and C2 (+0.040), indicating comparatively higher torque responses under the applied protocol, whereas the other genotypes showed parameter-dependent positive or negative BLUPs. Lina’s higher C1 and C2 BLUPs indicated greater relative dough consistency under the applied protocol. A possible contribution of glutenin composition or disulfide cross-linking may be hypothesized from the literature [23,24], but these characteristics were not measured directly. Nevertheless, Lina’s negative stability BLUP (−1.328) indicated lower relative mixing stability under the fixed hydration conditions. Snina showed positive BLUPs for the starch-related parameters (C3 = +0.235, C4 = +0.256, and C5 = +0.457), whereas Irchad showed negative BLUPs (C3 = −0.087, C4 = −0.202, and C5 = −0.558). The high C5 BLUP of Snina indicated greater consistency during cooling and may suggest a greater tendency toward starch reassociation [25]. However, amylose/amylopectin composition and staling behavior were not measured directly. Stability BLUPs also varied markedly: Ibtissam (+1.789) and Achtar (+1.305) showed the highest positive stability BLUPs compared with Kenz (−1.811) and Lina (−1.328), which showed negative stability BLUPs under the evaluated conditions. Malika showed positive BLUPs for several parameters, indicating a comparatively balanced rheological profile within the present dataset; however, its suitability for specific applications requires direct validation.

The analysis of interaction BLUPs (G × E) (Table S7) revealed substantial deviations centered around zero; for example, C1 ranged from −0.57 (Bandera–SEA) to +1.26 (Lina–MCH), C5 from −1.52 (Irchad–ANN) to +0.78 (Lina–MCH), and stability from −1.58 (Bandera–ANN) to +0.91 (Ibtissam–MCH). These variations indicated environment-specific deviations from the overall genotypic responses. Positive C1 and C2 interaction responses were observed at the MCH site: Lina had positive BLUP G × E values of C1 and C2 (+1.2556; +0.2800), and Irchad (+0.2749; +0.0563) and Faiza (+0.2679; +0.1551), whereas Ibtissam had a negative C1 interaction BLUP (−0.4902) but showed a positive stability interaction BLUP (+0.9139). Moreover, Lina, Irchad and V21 were found to have negative stability interaction BLUPs (−0.8435, −1.1529, and −1.1970, respectively), illustrating contrasting consistency and stability responses. This rheological pattern could potentially be associated with differences in glutenin organization and disulfide cross-linking, as suggested in the literature [5,26]. However, these structural characteristics were not measured in the present study. At the SEA site, the trend shifted more toward dough stability and final viscosity: Irchad (C4 = +0.2946; C5 = +0.4292), Snina (+0.1527; +0.2356), Arrehane (C5 = +0.2520; stability = +0.5324), and Faiza (C4 = +0.1675; C5 = +0.0845; stability = +0.5021) showed positive C4, C5 and/or stability interaction responses, while Bandera was strongly negative for C1–C5 at SEA (C1 = −0.5726; C5 = −0.7300) despite positive stability (+0.8713). The C3–C5 and γ responses may be consistent with differences in starch gelatinization, hot-gel stability and reassociation during cooling [25,27,28]. However, amylose/amylopectin composition and α-amylase activity were not measured, and the proposed mechanisms therefore remain hypotheses based on the literature. At the ANN site, the positive BLUP G × E values for C3 indicated higher relative C3 torque responses for Snina (+0.0798), Kenz (+0.0945), Arrehane (+0.0844), and Bandera (+0.1356). Simultaneously, positive stability interaction responses were observed in Khadija (stability = +0.7065), V21 (+0.5618), and Achtar (+0.6402); conversely, Lina (C5 = −0.7358) and especially Irchad (C5 = −1.5256) showed negative C5 interaction responses, indicating lower cooling-phase torque under ANN. These responses may be discussed in relation to amylose content, α-amylase activity and molecular reassociation [25]; however, these mechanisms were not measured in the present study.

The magnitude and structure of G × E interactions observed, particularly for C1, C5, and stability, are consistent with patterns reported in other Mediterranean and semi-arid wheat studies, thereby demonstrating environment-dependent variation in rheological responses within the tested network. In practice, the identification of genotypes with low BLUP G × E amplitude (Kenz, Najia, and Khadija) versus higher BLUP G × E amplitude (Lina and Irchad) provides a preliminary, protocol-dependent description of lower or higher inter-environment variation under fixed hydration. These classifications may change under sample-specific hydration and require validation across additional environments and cropping seasons before broader application.

### 3.4. Principal Component Analysis and Varietal Clustering

**Figure 2:**
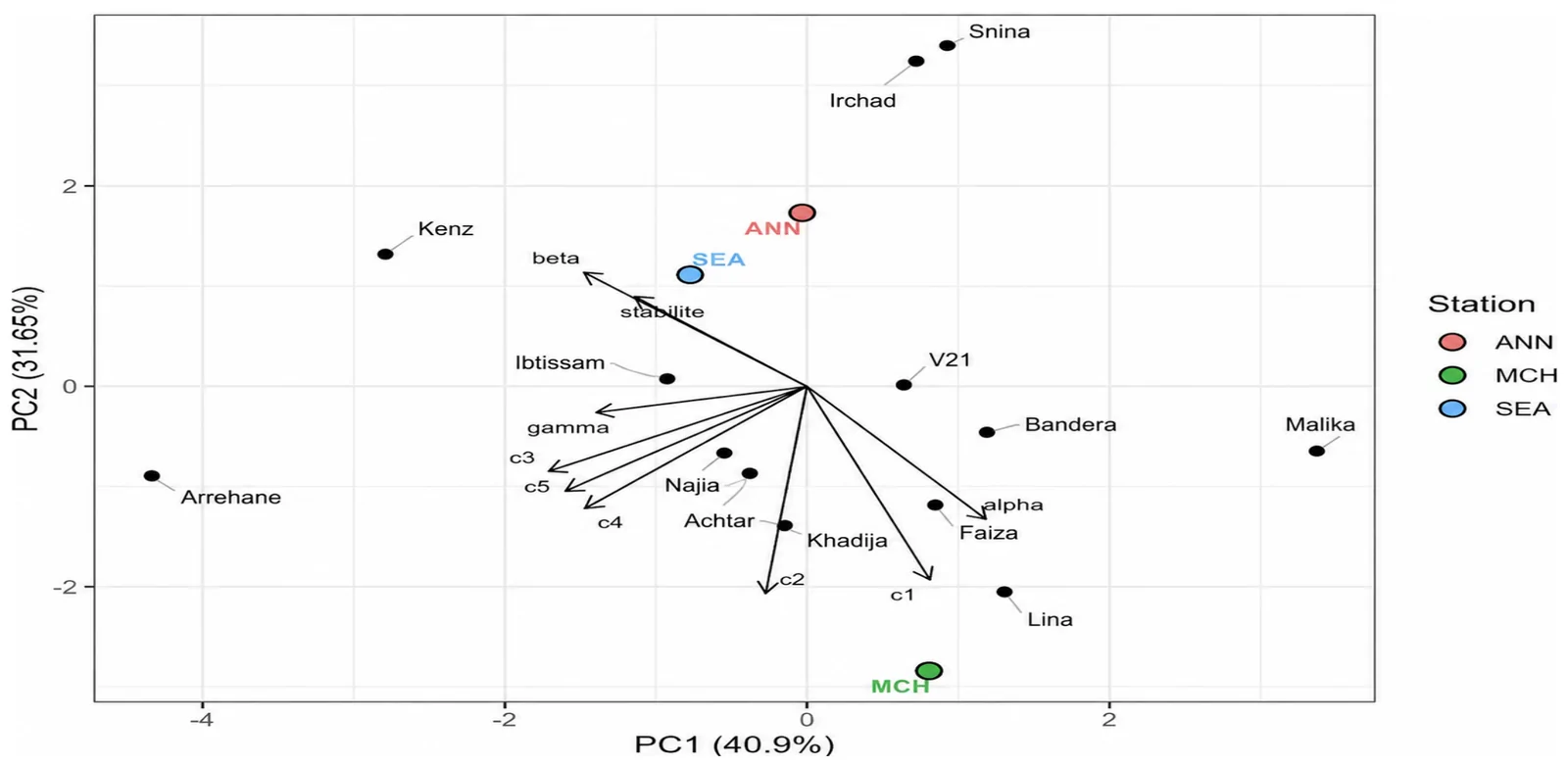
Principal component analysis of varieties based on rheological parameters and environments.

Principal component analysis (PCA) of the Mixolab^®^ 2 parameters explained 72.6% of the total variance (PC1 = 40.9%; PC2 = 31.65%) (Figure 2). This projection summarized the principal multivariate patterns among the evaluated varieties and environments and was interpreted as an exploratory representation of their relative rheological profiles.

The orientation of the vectors on the biplot showed associations among the measured rheological parameters. C1 and α were oriented toward the lower-right quadrant, whereas stability and β were oriented toward the upper-left quadrant. C3–C5 and γ were oriented toward the lower-left quadrant. These vector orientations describe associations among protocol-dependent Mixolab^®^ responses. Although C3–C5 and γ are related to starch behavior [5], the PCA alone does not demonstrate effects on protein strength, breadmaking quality, crumb texture or shelf life.

The station centroids also showed contrasting multivariate profiles. ANN and SEA were positioned on the positive side of PC2, closer to the stability and β vectors, whereas MCH was positioned in the lower-right quadrant, closer to the C1 and α vectors and opposite the stability vector. These positions indicate distinct station-level rheological profiles under the fixed hydration protocol.

The positions of the varieties on the biplot illustrated their relative multivariate profiles. Irchad and Snina were positioned in the upper part of the biplot and were separated from most other varieties mainly along PC2. Within the evaluated fixed hydration dataset, Kenz showed a comparatively high mixing tolerance response; its contribution to breadmaking performance must be confirmed through dedicated blending and baking trials. In the lower-left quadrant, Arrehane showed comparatively high C3–C5 and γ responses under fixed hydration; their implications for crumb firmness and staling require direct validation. Lina and Malika, located in the lower-right quadrant, showed comparatively higher consistency but lower stability responses under fixed hydration; no direct technological recommendation can be made from these responses alone. Finally, the varieties located near the PCA origin (Ibtissam, Najia, Achtar, Khadija, Bandera, and V21) showed rheological profiles closer to the overall multivariate average. The technological relevance of these profiles requires confirmation through dedicated formulation and baking experiments.

**Figure 3:**
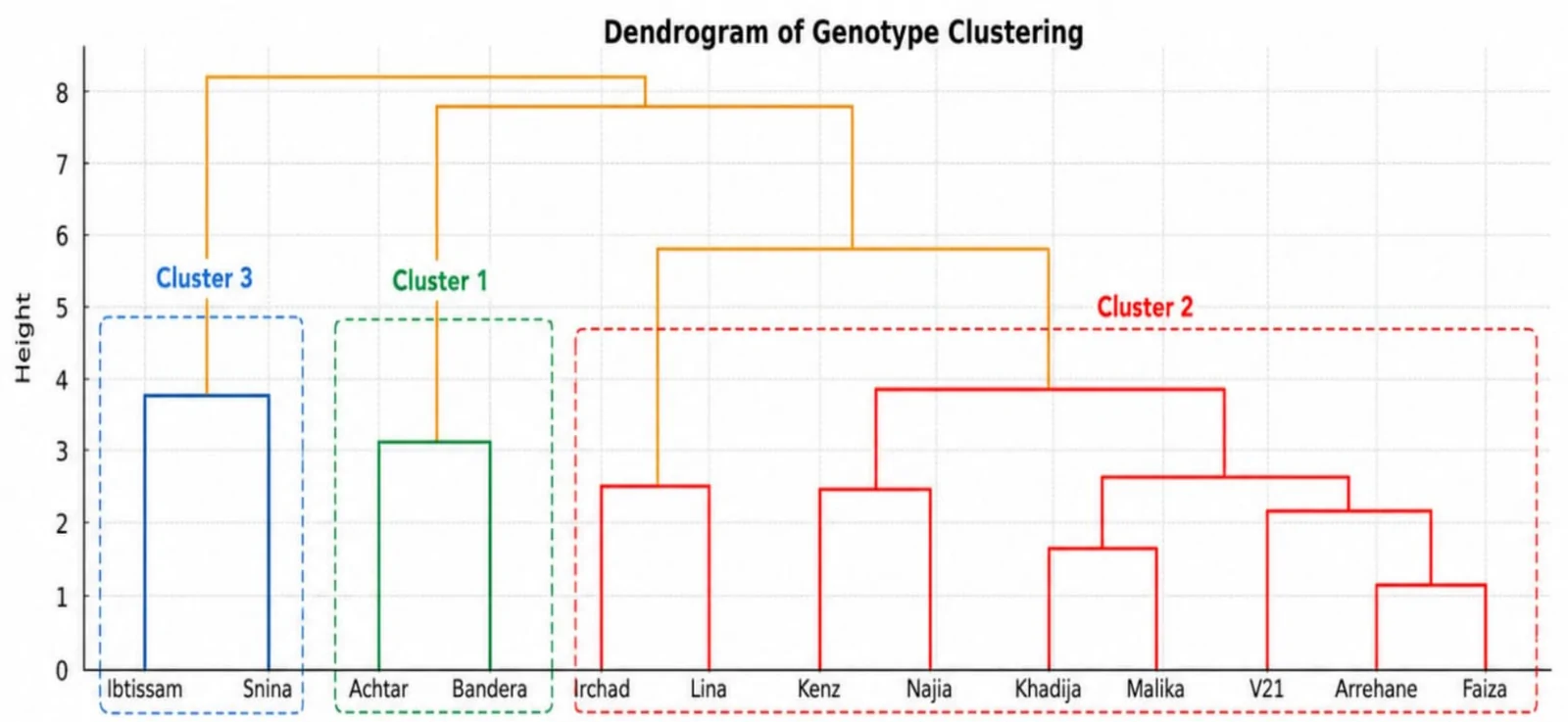
Hierarchical clustering dendrogram of the thirteen bread wheat varieties based on standardized mean Mixolab^®^ 2 rheological parameters using Ward’s method and Euclidean distances. The colored dashed boxes delineate the three identified clusters.

The hierarchical cluster analysis (Figure 3) revealed three well-defined groups of wheat varieties, each characterized by a distinct Mixolab^®^ profile. Mixolab^®^ records torque changes associated with protein weakening and starch transformations during mixing and heating [29]. The resulting parameters (C1–C5, α, β, γ, and stability) are functional rheological descriptors but do not directly measure baking quality.

The membership of each cluster and the corresponding mean ± standard deviation profiles for all Mixolab^®^ parameters are summarized in Supplementary Table S9.

Cluster 1, comprising Achtar and Bandera, showed a mean C1 value of 1.566 N·m and the lowest mean C2 and C3 values among the three clusters. According to the conventional interpretation of Mixolab^®^ parameters, this profile indicates comparatively lower torque responses during initial mixing, protein weakening and starch gelatinization [21,30]. Mixolab^®^ stability averaged 6.57 min, while the lower C4 and C5 values reflected lower torque responses during heating and cooling. These characteristics may have technological implications, but they do not demonstrate enzymatic sensitivity or suitability for a specific product without complementary biochemical and baking analyses.

Cluster 2, comprising Arrehane, Faiza, Irchad, Kenz, Khadija, Lina, Malika, Najia and V21, displayed the highest mean C1 and C2 values, intermediate C3–C5 values and the lowest Mixolab^®^ stability. This pattern reflects higher initial and protein-weakening torque values but lower mixing stability compared with the other clusters. The comparatively higher C4 and C5 values than those of Cluster 1 may indicate greater hot-gel and cooling-phase consistency [21,29,30]. Nevertheless, performance during fermentation and baking, as well as possible effects on crumb texture or staling, require direct validation.

Cluster 3, comprising Ibtissam and Snina, displayed the highest C3, C4, C5, β, γ and Mixolab^®^ stability values. These results may be consistent with higher torque responses during starch gelatinization, heating and cooling. However, because α-amylase activity, starch composition, breadmaking performance, crumb texture and storage behavior were not evaluated, the proposed technological implications remain hypotheses requiring further validation.

The standardized least-squares means (Table S8) showed station-specific differences in protocol-dependent Mixolab^®^ responses. These differences describe relative dough consistency, mixing tolerance, and heating–cooling torque responses under fixed hydration; they do not represent direct measurements of standard water absorption, biochemical composition, enzymatic activity, or end-product performance. They support relative rheological comparisons within the present dataset but do not establish suitability for breadmaking or other specific products [21,29,30].

### 3.5. The Inter-Environment Stability of the Varieties

The inter-environment stability analysis (Table 4), based on standardized G × E BLUP ranges, revealed contrasting protocol-dependent rheological responses among the thirteen varieties across the tested environments. Kenz, Najia, Khadija, Malika, and Ibtissam had the lowest mean width values, indicating comparatively smaller inter-environment variation within the evaluated fixed hydration dataset. Kenz, Najia, and Khadija occupied the first three positions in this preliminary ranking [31,32]. These positions should not be assumed to remain unchanged under the sample-specific hydration used in the standard ICC 173 procedure.

Conversely, Faiza, V21, Bandera, Lina and Irchad had higher mean and maximum width values, indicating greater inter-environment variation within the tested fixed hydration network.

Achtar, Snina and Arrehane showed intermediate levels of variation. Because the evaluation was restricted to three environments during a single cropping season, these rankings should be considered preliminary and should not be interpreted as definitive evidence of broad adaptation or used alone to formulate environment-specific varietal recommendations. Multi-year evaluations across a larger environmental network are required to confirm the observed rankings [31].

### 3.6. Study Limitations and Future Perspectives

All rheological measurements in the present study were performed on wholemeal flour, which retained the bran and germ fractions. These fractions can influence water distribution and absorption, interfere with gluten network development, and modify starch and enzymatic responses during the Mixolab^®^ heating–cooling cycle [33]. Consequently, the reported parameters reflect the behavior of the entire wheat kernel matrix and should not be directly compared with values obtained from refined bread flour. Interpretations related to bread volume, crumb texture, staling, or suitability for specific commercial breadmaking applications should therefore be regarded as potential technological implications rather than validated end-product performance. Direct validation using refined flour and controlled baking tests, including loaf volume and crumb texture measurements, is required in future studies.

Several limitations should be acknowledged. First, because the evaluation covered only one cropping season (2023/2024), location effects cannot be separated from the site-specific conditions prevailing during that particular season, and the temporal repeatability of the observed genotype and genotype × environment responses could not be assessed. Multi-year trials are therefore required to validate the environmental and genotypic rankings reported here. Second, only three environments were evaluated, which limits the precision of genotype × environment variance-component estimation and the broader generalization of the stability results. Third, the fixed 60% hydration protocol differed from the standard ICC 173 procedure, in which hydration is adjusted to obtain a C1 torque of 1.10 N·m. Consequently, the absolute values obtained in this study are not directly comparable with published Mixolab^®^ datasets generated using the standard variable hydration procedure. Because the same samples were not measured under both fixed hydration and standard target-torque conditions, it was not possible to determine whether genotype rankings would remain unchanged under sample-specific hydration. Fourth, complementary biochemical and quality measurements, including flour protein content, wet gluten, glutenin/gliadin composition, SDS sedimentation, damaged starch, Falling Number, amylose content and α-amylase activity, were not available. Mixolab^®^ responses associated with protein weakening and starch transformations should therefore be considered functional and indirect indicators rather than direct measurements of flour composition. Explanations involving gluten structure, disulfide cross-linking, starch composition, and enzymatic activity consequently represent literature-based hypotheses rather than mechanisms directly demonstrated by the present data. Fifth, although the field trials were established using a randomized complete block design, block identity was not retained in the final rheological dataset. Block effects were therefore not estimated separately, and the associated variability was included in the residual term. This limitation should be considered when interpreting genotype × environment interactions and genotypic stability. Future studies should incorporate these measurements together with direct baking and storage quality validation, compare fixed hydration and standard target-torque protocols, and retain block identity in the rheological dataset.

## 4. Conclusions

Under the fixed 60% hydration protocol, all nine Mixolab^®^ 2 parameters were significantly influenced by genotype (G), environment (E), and their interaction (G × E) (*p* < 0.001), confirming their responsiveness within the evaluated environmental network. Based on contributions to the corrected total sum of squares, E was particularly important for α (42.1%) and dough stability (33.8%), whereas G accounted for 43.8% of the variation in C5 and 35.2% in C4. The G × E interaction represented 50.8% of the variation in β and 38.3% in γ. Kenz, Najia, Khadija, Malika, and Ibtissam showed comparatively lower inter-environment variation in their protocol-dependent rheological profiles, with the lowest mean stability indices recorded for Kenz (0.402), Najia (0.412), and Khadija (0.423). Conversely, Lina (1.305) and Irchad (1.365) showed the greatest inter-environment variation under the same protocol. Because the same samples were not evaluated using the standard ICC 173 target-torque procedure, these rankings cannot be considered protocol-independent and may differ under sample-specific hydration. Given the evaluation of only three environments during one cropping season, these rankings should be considered preliminary and should not be used alone to formulate broad or environment-specific varietal recommendations. Furthermore, it cannot be determined from the present data whether the observed genotypic rankings would remain the same under the standard variable hydration protocol, and the results should therefore be interpreted as specific to the fixed hydration conditions applied in this study. The multi-environment analytical framework provided an exploratory description of rheological variability and differentiated the evaluated genotypes according to their relative stability profiles under a common hydration level. Combining Mixolab^®^ 2 parameters with mixed linear models and multivariate analyses may support preliminary wheat quality comparisons under fixed hydration conditions. Future work should expand this framework to a greater number of environments and cropping seasons, compare fixed hydration and standard target-torque protocols, and develop predictive indicators linking dough rheology more directly to final product quality.

## Figures and Tables

**Table 1:** Moroccan bread wheat varieties evaluated in this study, their breeders, and year of registration.

Code	Variety	Breeder	Year of Registration
1	Irchad	INRA-MAROC	2023
2	Ibtissam	INRA-MAROC	2022
3	Snina	INRA-MAROC	2017
4	Najia	FLORIMOND DESPREZ	2010
5	Kenz	INRA-MAROC	1988
6	Malika	INRA-MAROC	2016
7	Faiza	FLORIMOND DESPREZ	2010
8	Achtar	INRA-MAROC	1988
9	Lina	INRA-MAROC	2020
10	Khadija	INRA-MAROC	2012
11	Bandera	SERASEM	2010
12	Arrehane	INRA-MAROC	1996
13	V21	INRA-MAROC	Under registration

**Table 2:** Fixed-effects ANOVA and relative contributions of genotype, environment and genotype × environment interaction to Mixolab^®^ parameters.

Parameter	Genotype F (*p*-Value)	G Contribution (%)	Environment F (*p*-Value)	E Contribution (%)	G × E F (*p*-Value)	G × E Contribution (%)	Residual Contribution (%)
C1	22.730 *** (<2 × 10^−16^)	27.7	158.110 *** (<2 × 10^−16^)	32.1	13.230 *** (<2 × 10^−16^)	32.3	7.9
Stability	10.427 *** (6.42 × 10^−12^)	30.8	68.692 *** (<2 × 10^−16^)	33.8	2.741 *** (4.36 × 10^−4^)	16.2	19.2
C2	6.090 *** (2.09 × 10^−7^)	22.7	38.285 *** (2.61 × 10^−12^)	23.8	3.936 *** (2.31 × 10^−6^)	29.3	24.2
C3	7.462 *** (5.96 × 10^−9^)	32.1	18.900 *** (2.03 × 10^−7^)	13.6	3.053 *** (1.08 × 10^−4^)	26.3	28.0
C4	11.193 *** (1.30 × 10^−12^)	35.2	24.555 *** (5.36 × 10^−9^)	12.9	4.998 *** (3.08 × 10^−8^)	31.5	20.5
C5	55.310 *** (<2 × 10^−16^)	43.8	125.260 *** (<2 × 10^−16^)	16.5	21.800 *** (<2 × 10^−16^)	34.5	5.1
α	4.267 *** (3.68 × 10^−5^)	14.2	76.119 *** (<2 × 10^−16^)	42.1	3.323 *** (3.27 × 10^−5^)	22.1	21.6
β	7.583 *** (4.41 × 10^−9^)	23.9	9.342 *** (2.31 × 10^−4^)	4.9	8.062 *** (7.59 × 10^−13^)	50.8	20.5
γ	8.111 *** (1.21 × 10^−9^)	23.9	38.049 *** (2.93 × 10^−12^)	18.7	6.513 *** (1.18 × 10^−10^)	38.3	19.1

F-statistics were calculated using 12, 2 and 24 numerator degrees of freedom for genotype, environment and genotype × environment interaction, respectively, and 78 residual degrees of freedom. Contributions represent the percentage of the total corrected sum of squares attributable to each source. Percentages may not sum exactly to 100 because of rounding. G × E, genotype × environment interaction; *** *p* < 0.001;

**Table 3:** Station-level summary of Mixolab^®^ 2 responses under fixed 60% hydration.

Parameter	MCH	SEA	ANN
C1 (N·m)	2.310 ± 0.578	1.755 ± 0.291	1.705 ± 0.196
C2 (N·m)	1.013 ± 0.177	0.844 ± 0.201	0.745 ± 0.125
C3 (N·m)	1.752 ± 0.217	1.569 ± 0.218	1.790 ± 0.186
C4 (N·m)	1.625 ± 0.197	1.830 ± 0.259	1.499 ± 0.455
C5 (N·m)	3.086 ± 0.425	3.060 ± 0.468	2.489 ± 0.876
Stability (min)	4.851 ± 1.925	8.086 ± 1.365	5.717 ± 1.695
α (N·m ·min^−1^)	−0.120 ± 0.023	−0.088 ± 0.012	−0.083 ± 0.009
β (N·m ·min^−1^)	0.185 ± 0.090	0.252 ± 0.123	0.228 ± 0.125
γ (N·m ·min^−1^)	−0.100 ± 0.103	−0.016 ± 0.042	−0.053 ± 0.026

Values are means ± standard deviations calculated across the thirteen genotype means within each station (n = 13). All measurements were obtained under fixed 60% hydration. Therefore, C1 represents the observed dough consistency rather than consistency standardized to the ICC 173 target torque of 1.10 N·m. Complete genotype-specific means and statistical comparisons are provided in Supplementary Tables S2–S4.

**Table 4:** Ranking of varieties according to relative inter-environment stability under fixed 60% hydration.

Variety	Width_Mean	Width_Med	Width_Max	Rank_Stability
Kenz	0.402	0.178	1.817	1
Najia	0.412	0.277	1.517	2
Khadija	0.423	0.282	1.65	3
Malika	0.456	0.266	1.85	4
Ibtissam	0.471	0.289	1.8	5
Achtar	0.532	0.268	1.462	6
Snina	0.539	0.236	3.2	7
Arrehane	0.593	0.126	3.85	8
Faiza	0.869	0.504	4.85	9
V21	0.973	0.317	5.7	10
Bandera	1.061	0.491	6.133	11
Lina	1.305	0.736	5.3	12
Irchad	1.365	0.442	6.25	13

## Data Availability

The original contributions presented in this study are included in the article/Supplementary Materials. Further inquiries can be directed to the corresponding author.

## Supplementary Materials

The following supporting information can be downloaded at: https://www.mdpi.com/article/10.3390/foods15183238/s1, Table S1. Agro-climatic, edaphic and field-management characteristics of the three experimental environments during the 2023/2024 cropping season. Table S2. Mean performance of thirteen Moroccan bread wheat varieties for nine rheological parameters at Merchouch (MCH) station according to the least significant difference (LSD) test at *p* < 0.05. Table S3. Mean performance of thirteen Moroccan bread wheat varieties for nine rheological parameters at Sidi El Aidi (SEA) station according to the least significant difference (LSD) test at *p* < 0.05. Table S4. Mean performance of thirteen Moroccan bread wheat varieties for nine rheological parameters at Annoceur (ANN) station according to the least significant difference (LSD) test at *p* < 0.05. Table S5. Pearson’s correlation coefficient of rheological parameters measured by Mixolab 2 in three environments for Moroccan bread wheat varieties. Table S6. Genotypic best linear unbiased predictions (BLUP-G) for nine Mixolab® rheological parameters across the three environments. Table S7. Genotype-by-environment best linear unbiased predictions (BLUP-GE) for nine Mixolab® rheological parameters. Table S8. Overall least-squares means of thirteen Moroccan bread wheat varieties for nine Mixolab® rheological parameters across the three environments. Table S9. Cluster membership and mean Mixolab® parameter profiles of the evaluated bread wheat varieties.
